# X‐ray scattering reveals disordered linkers and dynamic interfaces in complexes and mechanisms for DNA double‐strand break repair impacting cell and cancer biology

**DOI:** 10.1002/pro.4133

**Published:** 2021-06-05

**Authors:** Michal Hammel, John A. Tainer

**Affiliations:** ^1^ Molecular Biophysics and Integrated Bioimaging Lawrence Berkeley National Laboratory Berkeley California USA; ^2^ Department of Cancer Biology University of Texas MD Anderson Cancer Center Houston Texas USA; ^3^ Department of Molecular and Cellular Oncology University of Texas MD Anderson Cancer Center Houston Texas USA

**Keywords:** backbone conformation, cancer, DNA repair, dynamic structures, functional dynamics, genome stability, quantitative flexibility, supramolecular structures, unstructured regions

## Abstract

Evolutionary selection ensures specificity and efficiency in dynamic metastable macromolecular machines that repair DNA damage without releasing toxic and mutagenic intermediates. Here we examine non‐homologous end joining (NHEJ) as the primary conserved DNA double‐strand break (DSB) repair process in human cells. NHEJ has exemplary key roles in networks determining the development, outcome of cancer treatments by DSB‐inducing agents, generation of antibody and T‐cell receptor diversity, and innate immune response for RNA viruses. We determine mechanistic insights into NHEJ structural biochemistry focusing upon advanced small angle X‐ray scattering (SAXS) results combined with X‐ray crystallography (MX) and cryo‐electron microscopy (cryo‐EM). SAXS coupled to atomic structures enables integrated structural biology for objective quantitative assessment of conformational ensembles and assemblies in solution, intra‐molecular distances, structural similarity, functional disorder, conformational switching, and flexibility. Importantly, NHEJ complexes in solution undergo larger allosteric transitions than seen in their cryo‐EM or MX structures. In the long‐range synaptic complex, X‐ray repair cross‐complementing 4 (XRCC4) plus XRCC4‐like‐factor (XLF) form a flexible bridge and linchpin for DNA ends bound to KU heterodimer (Ku70/80) and DNA‐PKcs (DNA‐dependent protein kinase catalytic subunit). Upon binding two DNA ends, auto‐phosphorylation opens DNA‐PKcs dimer licensing NHEJ via concerted conformational transformations of XLF‐XRCC4, XLF–Ku80, and LigIV^BRCT^–Ku70 interfaces. Integrated structures reveal multifunctional roles for disordered linkers and modular dynamic interfaces promoting DSB end processing and alignment into the short‐range complex for ligation by LigIV. Integrated findings define dynamic assemblies fundamental to designing separation‐of‐function mutants and allosteric inhibitors targeting conformational transitions in multifunctional complexes.

## 1 INTRODUCTION

Macromolecular flexibility, unstructured linkers, dynamic conformations, and metastable complexes are essential functional aspects of DNA damage response (DDR) regulatory mechanisms. This finding has implications for defining their structural biochemistry underlying genome stability, cancer avoidance, and outcome to cancer therapies. Macromolecular X‐ray crystallography (MX) and cryo‐electron microscopy (cryo‐EM) are powerful methods for determining atomic positions in protein–protein and protein‐DNA complexes to provide precise atomic structures with some information on flexible regions. Yet, systematic analyses of their accuracy show these detailed structures can be too rigid versus functional solution structures.^1^, ^2^, ^3^, ^4^, ^5^, ^6^, ^7^, ^8^, ^9^, ^10^, ^11^, ^12^ Therefore for DNA repair and damage responses ranging from oxidized base repair to DNA double‐strand break (DSB) repair (DSBR), we have found that accurate measures of flexibility, conformational change, and dynamic complexes from small‐angle X‐ray scattering (SAXS) are often important for understanding and dissecting multifunctional mechanisms, as exemplified by the intrinsically disordered tail of Nei Like DNA Glycosylase 1 (NEIL1) acting in efficient oxidized base repair^13^, ^14^ and by ATP‐driven RAD50 assembly and conformational states acting in the homology‐directed repair (HDR) of DSBR.^15^, ^16^, ^17^, ^18^, ^19^, ^20^

Furthermore, many crystal structures have trimmed N and C‐termini (due to their flexibility), and this need for low conformational heterogeneity merits complementary SAXS studies to examine the function of full‐length proteins and complexes. Even the crystal structure of the direct damage reversal ALKBH3 enzyme, which reverses alkylation damage to restore the native DNA damage, required removal of its flexible N‐terminus.^21^ For Rad51, which acts in HDR, the functionally flexible polymerization motif lies in the linker region between domains; this made it so challenging to see correct assemblies that a thermophile was employed to define the first intact Rad51 structure and assembly.^22^ Fortunately, SAXS provides an accurate measure of the solution ensemble plus the means to examine unstructured regions and to assess conformational changes and assembly states critical to DNA repair activities; this is invaluable for complementing many X‐ray, cryo‐EM, and NMR structures.^1^, ^2^, ^16^, ^23^, ^24^, ^25^, ^26^

As a central facet of their function, DNA repair proteins face the difficulty of differentiating their target DNA damage from the much more populated undamaged DNA.^14^, ^27^, ^28^ To accomplish damage recognition, they often distort the DNA, such as damaged nucleotide flipping in base excision repair.^29^, ^30^, ^31^, ^32^, ^33^ They also use steric molds to check for the presence of damage or another specific characteristic of their substrate.^28^ For example, glycosylases and apurinic/apyrimidinic endonucleases use phosphate backbone pinching to test for disrupted base stacking that allows for flipping out of the nucleotide or phosphodiester into damage‐specific molds.^34^, ^35^, ^36^ Indeed, stable binding to flipped out alkylated DNA bases can mark alkylated base damage and enable a handoff from base to nucleotide excision repair for efficient damage removal.^4^, ^37^ For excision enzymes, only if the flipped‐out DNA can be retained is activity enabled. As a prototypic example, the structure‐specific flap endonuclease FEN1 uses DNA distortion, phosphate steering, and DNA‐induced protein conformational changes to validate the presence of a 5′ flap plus a 3′ 1‐nucleotide flap within dsDNA before an incision is licensed 1‐nucleotide into the dsDNA at the 5′ flap.^38^, ^39^ Conversely the structure‐specific nuclease EXO5 uses order‐to‐disorder of an active channel cross‐over helix to specifically thread and processes 5′ ends to restart inverted stalled replication forks.^40^ The nuclease MRE11 complex with RAD50 ATPase similarly undergoes dramatic conformational changes that allow validation of dsDNA ends for HDR.^18^ These protein and DNA conformational changes enable repair complexes to find and validate DNA damage versus normal B‐DNA, which provides stability and base protection,^27^, ^41^ and to examine open chromatin areas associated with both increased oxidative damage and gene expression.^8^, ^14^, ^42^

To coordinate repair and reduce the risk of toxic intermediates, repair enzymes are often product inhibited and only release a product when the following enzyme is present. Indeed, there is growing appreciation for the metastable assemblies of DNA repair enzymes. In double‐strand break repair (DSBR), there is a temporally coordinated assembly of proteins at DNA ends.^10^, ^18^, ^43^, ^44^, ^45^ The DNA ends to be rejoined will need to be protected, held to keep a DSB from becoming a chromosome break, processed to make both ends suitable for ligation, and aligned for joining: this requires flexibility and dynamic assemblies in DSBR proteins and especially in their key scaffold proteins such as XRCC1 that enables alternative end‐joining for DSBR and replication restart.^1^, ^46^ Yet even in dynamic nucleotide excision repair (NER) assemblies, the extreme precision of the excised oligonucleotide supports TFIIH‐based licensing and ruler features that strictly dictate when and where the incision sites occur relative to the lesion.^47^ We will show here that the dynamic phosphoinositide 3‐kinase‐related DNA‐PK catalytic subunit (DNA‐PKcs) has an analogous licensing and ruler function in non‐homologous end joining (NHEJ).

From the above considerations, it is clear that dynamic features and assemblies are essential elements in DNA repair functions that almost paradoxically enable extreme precision in DNA damage recognition and repair. Efficiency and precision surprisingly do not primarily emerge from the relatively rigid lock‐and‐key principle. Rather we argue that they largely arise from a flexible conformational control principle whereby domain rotations, plastic deformations, and disorder–order transitions in multifunctional macromolecular machines enable specificity via structurally‐encoded inducible complementarity for repair complexes and damaged DNA. So to understand DSBR mechanisms, it is critical to determine solution conformations and assembly states. We find that SAXS is an enabling technique to structurally characterize protein conformations in solution under near‐physiological conditions with high‐throughput and super‐resolution.^48^, ^49^ Fortunately, collecting SAXS data are straightforward and essentially available to any scientist who has protein, RNA, or DNA^1^, ^4^, ^8^, ^23^, ^50^ due to the availability of synchrotron beamline facilities such as SIBYLS.^51^ Importantly, SAXS results readily complement and enhance structural results from cryo‐EM, MX, NMR, and computational modeling, so we see SAXS as a premier technique for integrative structural biology.^2^, ^48^, ^51^, ^52^, ^53^, ^54^, ^55^, ^56^ Thus, combining data from solution scattering with atomic resolution structures can address how specific complexes, conformations, and flexibility drive biological processes such as DSBR.^54^, ^56^, ^57^ Although as with any biophysical technique SAXS has its inherent limitations,^55^, ^58^ there is typically sufficient information from most samples to provide objective quantitative data on assembly and flexibility.^56^, ^59^ Additionally, SAXS profiles can be efficiently calculated from atomistic models and directly matched to experimental data.^52^, ^53^, ^60^, ^61^ As a result, multistate data‐based models^52^, ^53^, ^56^, ^62^, ^63^ that incorporate dynamic rearrangements (such as domain motions, transient complexation, and unfolded regions) can be robustly determined by SAXS‐based atomistic modeling. In fact, although DNA repair can involve the dynamic assembly of supramolecular machines and metastable complexes rather than a strictly linear pathway,^64^ we have learned much even from core domains and complexes when we include knowledge of their protein and DNA conformational changes and consider them as components of molecular machines.^65^, ^66^, ^67^, ^68^, ^69^

This treatise will examine NHEJ structural assembles and their multifunctional dynamicity as determined by SAXS measurements combined with cryo‐EM and MX structures. We focus on NHEJ as an exemplary and critical DSBR system: it is the major machine for the repair of double‐stranded DNA breaks (DSBs) including ionizing radiation (IR)‐induced DSBs in human cells.^70^, ^71^ The NHEJ initiation complex is DNA‐PK, which consists of the Ku70 (XRCC6) and Ku80 (XRCC5) heterodimer (KU) and the DNA‐dependent protein kinase catalytic subunit (DNA‐PKcs). Other critical component proteins are the scaffolding proteins XRCC4 (X‐ray repair cross‐complementing 4) and XRCC4‐like factor XLF plus DNA ligase IV (LigIV). The KU heterodimer, which binds DNA ends, detects the DSB and recruits DNA‐PKcs to form the initial DNA‐PK assembly on DNA ends, also called the presynaptic complex.^70^ Importantly this presynaptic complex protects and holds two DNA ends in concert with core scaffold proteins XRCC4‐XLF and LigIV plus PAXX (PAralog of XRCC4 and XLF), which can be functionally replaced by lncRNA (long noncoding RNA) LINP1 in NHEJ.^50^ Together the DNA‐PK complex, XRCC4‐XLF scaffold proteins, and LigIV form the long‐range (LR) complex, as the two DNA ends are protected but not processed or aligned. Further DNA end processing can be required to remove damaged DNA and non‐ligatable end groups at the termini of the DSB to facilitate ligation. This processing requires access to the DNA ends and may involve polynucleotide kinase/phosphatase (PNKP), aprataxin and PNKP related protein (APLF), DNA polymerases, and the hairpin specific nuclease Artemis.^71^, ^72^, ^73^ For LigIV to join the DNA ends requires dynamic interface and assembly changes to form a short‐range (SR) synaptic complex wherein DNA ends are aligned but still bridged by XRCC4‐XLF and LigIV, which can be further stabilized by APLF, PAXX, or LINP1 scaffolds.

In recent cases where cryo‐EM provided near‐atomic resolution, integration of high‐resolution structures of the components^74^, ^75^ or partial assemblies^76^ into the cryo‐EM maps enabled the reconstruction of breakthrough atomistic models for the LR and SR synaptic complexes.^10^ Notably, NHEJ requires dynamic mechanisms enabled by flexible complexes, but to create tractable samples for cryo‐EM analysis, a crosslinking agent was required to stabilize the complexes. Such crosslinking may limit assessment of flexibility but also implies the complexes are functionally dynamic. Indeed, significant allosteric transitions are expected for function, including (a) transition from DSB recognition by KU to form the LR presynaptic complex (by recruiting DNA‐PKcs, XRCC4, and XLF), (b) access for DNA end processing by Artemis nuclease and PNKP kinase/phosphatase within XLF‐XRCC4 scaffolded DNA ends, and (c) ligation by LigIV enabled in the SR complex. Here we elucidate dynamic NHEJ complexes by combining comprehensive solution‐state SAXS measurements with available higher resolution static structures of NHEJ complexes to provide an integrated perspective on functionally relevant solution behavior of NHEJ assemblies in DSBR. The presented analysis provides new insights, suggests corrections for some misconceptions, and provides resolutions for controversies about the roles of DNA‐PKcs and its partners in NHEJ.

## 2 DNA‐PK FUNCTIONAL PLASTICITY ORCHESTRATES NHEJ INITIATION

In vitro it is possible to show NHEJ without the DNA‐PK catalytic subunit DNA‐PKcs.^77^, ^78^ These data reveal that DNA‐PKcs is not an essential part of the short‐range synaptic complex for joining the DNA ends by LigIV. It was also thought that genetics and evolution supported the idea that DNA‐PKcs were phylogenetically recent, but this idea has been corrected by recent comprehensive sequence analyses.^44^ Furthermore, we know that the DNA‐dependent kinase subunit DNA‐PKcs is critical for orchestrating NHEJ in response to ionizing radiation and other DSB‐causing events in cells.^44^ Fortunately, structural biology provides insight on DNA‐PK functions not revealed by end‐joining assays. DNA‐PKcs has key protein interfaces in at least one of the two critical and distinct synaptic states prior to DSB ligation in NHEJ. In the first DSB response, KU and DNA‐PKcs (the DNA‐PK complex) provide a long‐range tether for DNA ends at a distance where they are protected from processing: this is the long‐range (LR) synaptic complex.^79^, ^80^ DNA‐PK plus XRCC4, XLF, and LigIV form this LR complex, in which the DNA ends are protected but held ~115 Å apart.^10^ In this and the following sections, we will argue that viewing NHEJ as if it is a linear pathway, rather than a supramolecular machine as we do herein, will result in confusions and misconceptions regarding the functional importance of components and activities.

**FIGURE 1:**
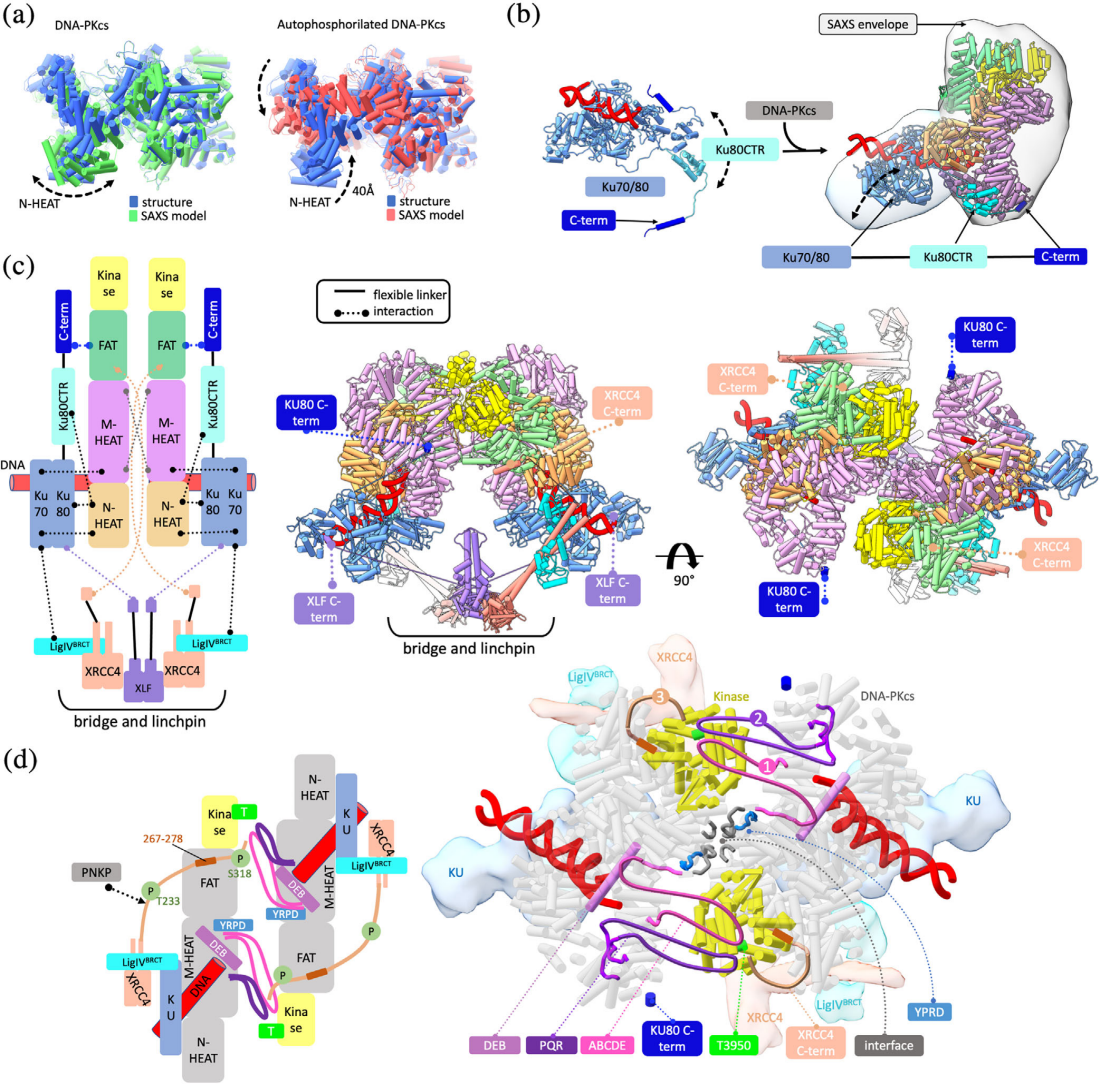
Formation of the long‐range synaptic complex from dynamic components, modular interfaces, and flexible scaffolding. (a) Inherent dynamicity of DNA‐PKcs HEAT region and its rearrangement during the autophosphorylation. The multi‐state model used to match experimental SAXS curves of DNA‐PKcs, and auto‐phosphorylated DNA‐PKcs indicates significant motion of HEAT domains (taken from Reference 2). Atomistic models are colored according the legend. (b) Left panel: Inherent dynamicity of KU80CTR and KU80 C‐terminus as visualized in SAXS‐based multi‐state model of KU‐DNA complex.^2^ Right panel: The cryo‐EM structure of DNA‐PK^76^ is superimposed onto the multiphase SAXS envelope of DNA‐PK taken from.^25^ A mismatch between the SAXS envelope and cryo‐EM structure suggests conformational variability of KU‐DNA in the absence of XRCC4‐XLF‐XRCC4 “bridge” and “linchpin” that stabilize DNA‐PK assembly in the LR synaptic complex (see panel c). The KU and DNA‐PKcs domains are colored according to the schematic representation shown in panel c. The schematic representation of KU highlights the extension of the KU80CTR and KU80 C‐terminus that undergoes upon recruiting the DNA‐PKcs. (c) Cryo‐EM structure of the LR synaptic complex.^10^ Left panel: The LR complex's schematic representation highlights the importance of XLF, XRCC4, and KU flexible tethers to juxtaposition components in the synaptic complex. Right panel: Two orthogonal views of the structural model of the LR complex. The extension of the XLF C‐terminus, XRCC4 C‐terminus, and KU80CTR from the core is highlighted. Complex components are colored according to the schematic representation. Solid and dotted lines represent the flexible tethers or components interactions, respectively. (d) DNA‐PK activation proceeds through multiple distinct steps. Left panel: The LR complex's schematic representation highlights the importance of DNA‐PKcs dimer interface to juxtaposition disordered ABCDE, PQR, and XRCC4C‐terminal phosphorylation site into the proximity of kinase active site (T3950 residue colored in green). The schematic representation also highlights XRCC4 C‐terminus (267–278) interacting with DNA‐PKcs FAT domain and PNKP interaction with disordered XRCC4 C‐terminus that is controlled by CK2 phosphorylation of XRCC4 T233 residue. Right panel: The structural model of the LR complex. The schematical representation of the extension of the ABCDE, PQR, and XRCC4C‐terminal phosphorylation sites are highlighted in the DNA‐PKcs structure (gray). Phosphorylation sites are also numbered based on our hypothetical model of multistep DNA‐PKcs activity. Blockage and DNA strand (red) separation by DEB helix (violet) is highlighted. The DNA‐PKcs dimer interface formed between 896–903 and 2,569–2,585 DNA‐PKcs loops (dark ray) is supported by highly conserved YRPD motives (blue). Other complex components are colored according to the schematic representation

In the LR synaptic complex, DSB detection and DNA end protection by KU is followed by recruitment of the DNA‐PKcs, which will subsequently undergo DNA stimulated auto‐phosphorylation to regulate repair progression.^81^, ^82^, ^83^ Multiple important DNA‐PKcs structures were solved using MX^75^, ^84^ and cryo‐EM.^76^, ^85^, ^86^, ^87^ Together with previously reported cryo‐EM low‐resolution molecular envelopes,^88^, ^89^ these data suggest that the DNA‐PKcs M‐HEAT and N‐HEAT domains are flexibly attached to the “head” region containing the FAT and kinase domains (Figure 1). The HEAT domains are formed by repeats of two anti‐parallel α‐helices and two turns arranged about a common axis; flexible inter‐unit loops link these repeats. Their plasticity allows them to act in conformational allosteric movements during auto‐phosphorylation^81^, ^87^ and rearrange upon interaction with the KU‐DNA complex.^76^, ^85^, ^87^ Indeed, DNA‐PKcs in solution undergoes much larger allosteric transitions than shown in cryo‐EM or MX structures.^2^, ^81^ By employing SAXS, the static structure of DNA‐PKcs was found to adopt dynamic multistate functional conformations with HEAT domain flexibility visualized experimentally in solution.^2^

By assuming that the DNA‐PKcs domain movements (Figure 1a) are inter‐dependent, conformational sampling by normal mode analysis (NMA) was explored.^90^ SAXS profiles were calculated from atomistic models and directly matched to experimental data. Conformational sampling was followed by selecting a multistate data‐based model.^53^, ^56^, ^62^ A two‐state DNA‐PKcs model significantly improved fit to the SAXS data and showed extensive rearrangement of the HEAT region in solution^2^ (Figure 1b). DNA‐PKcs plasticity results from its architectural integration of multiple local stretch and twist changes of HEAT repeats.^87^ Such movements within individual HEAT‐solenoids may function as spring‐like energy, which transforms the conformational signal into the kinase domain upon the interaction with KU‐DNA, followed by DNA‐PKcs autophosphorylation and its release.^81^, ^82^, ^91^ Importantly, the SAXS‐model of auto‐phosphorylated DNA‐PKcs showed large (∼40 Å) displacements of both the N‐ and M‐HEAT regions leading to the closure of the aperture between these domains^2^ (Figure 1b). Notably, these domain motions are large as the HEAT domain rearrangement was observed in cryo‐EM upon recruiting KU‐DNA.^10^, ^76^, ^85^, ^87^ Rearrangement of the entire HEAT region upon the autophosphorylation suggested inaccessibility of the KU/N‐HEAT binding site. By making the KU/N‐HEAT binding site inaccessible, we hypothesized that DNA‐PKcs is largely released from KU‐DNA by autophosphorylation to allow processing enzymes like LigIV and PNKP to access an aligned DSB and a short‐range (SR) synaptic DNA complex held by an XRCC4‐XLF flexible bridge without DNA‐PKcs.^79^ This idea has been supported and extended by recent structures discussed below.

## 3 KU‐BOUND DNA ENDS ARE TETHERED BY A FLEXIBLE XRCC4‐XLF‐XRCC4 BRIDGE AND LINCHPIN

To initiate NHEJ, KU binds to DNA ends and recruits DNA‐PKcs to form the DNA‐PK complex. The KU crystal structure and its complex with DNA were solved over two decades ago.^74^ However, the flexibility of the Ku80 C‐terminal region (KU80CTR) and KU80CTR C‐terminal helix,^81^ responsible for DNA‐PKcs interaction,^92^ prevents the visualization of full‐length KU by MX or cryo‐EM. Fortunately, SAXS‐based measurements and modeling identify a preferentially close interaction between the flexibly linked KU80CTR region and the KU core (Figure 1c). Significant improvement in the SAXS fit was achieved by selecting the two‐state model that included conformers with detached KU80CTR domain (~30 Å distance) and large distancing of the KU80 C‐terminal region (CTR) helix. When KU is bound to DNA‐PKcs to form DNA‐PK assembly, the KU80CTR region is far more extended from the KU core (~60 Å)^85^, ^87^ than in the free state as identified by SAXS (Figure 1c).^2^, ^50^ The C‐terminal helix of KU80CTR is even more distant (~80 Å) from the KU core.^75^, ^85^, ^87^ Thus, the KU80CTR domain, including the KU80CTR C‐terminus, must undergo a large displacement during KU interaction with DNA‐PKcs. Such a dramatic rearrangement is enabled by the flexible ~60 residue long KU80CTR linker.^2^, ^92^

The flexibly tethered KU80CTR C‐terminus helix must find its binding site near the “PQR” autophosphorylation cluster^10^, ^75^, ^85^, ^87^ (Figure 1b,c). Thus, initial tethering is followed by recruiting the KU core to the N‐HEAT binding site, allowing insertion of the DNA end into the M‐/N‐HEAT aperture (Figure 1c). SAXS revealed the relatively compact arrangement of the KU80CTR domain in the presence of DNA (Figure 1c).^2^ Thus, the KU80CTR “arm”‐like extension upon DNA‐PKcs complexation is promoted by interaction between the KU80CTR C‐terminus and the M‐HEAT domain^75^, ^85^, ^87^ rather than by DNA binding. Dimers of DNA‐PK are observed in low‐resolution SAXS envelopes^2^, ^81^ and low‐resolution cryo‐EM studies.^93^ Surprisingly, a different dimer arrangement of DNA‐PK was reported by the cryo‐EM structure of DNA‐PK at ~4 Å resolution that reveals a dimer mediated by domain swap of the KU80CTR C‐terminal helix^85^; yet, a more recent cryo‐EM study unveils the likely biologically active DNA‐PK dimer assembly^10^ (Figure 1c).

Key synaptic complex protein partners XRCC4, XLF, and LigIV are independently recruited to KU‐bound DNA ends, and each of these has some end‐bridging activity.^94^, ^95^, ^96^, ^97^, ^98^ The reconstructed DNA‐PK‐XRCC4‐LigIV‐XLF assembly shows symmetric folding between two loops from each copy of DNA‐PKcs.^10^ Loop 2,569–2,585 interacts with the evolutionarily conserved YRPD motif.^44^ However, most notably, the DNA‐PK dimer is extensively stabilized through XRCC4‐XLF‐XRCC4, which acts as both flexible “bridge” and “linchpin”^10^ (Figure 1c). Prior SAXS data show that the XRCC4‐XLF‐XRCC4 “bridge” also forms in the absence of DNA‐PK when XRCC4 is complemented with the LigIV^BRCT^ domain (see Figure 4b).^99^ These results establish the flexible bridge's structural integrity while also supporting the disorder of the conserved XRCC4 C‐terminus, enabling its flexible functional interactions with DNA‐PK. Indeed, the cryo‐EM structure of the LR complex shows the interaction between far‐reaching XRCC4 C‐terminal region 267–278 and DNA‐PKcs FAT domain, where the XRCC4C‐terminal phosho‐site can reach the catalytic domain and activate DNA‐PKcs.^10^

**FIGURE 2:**
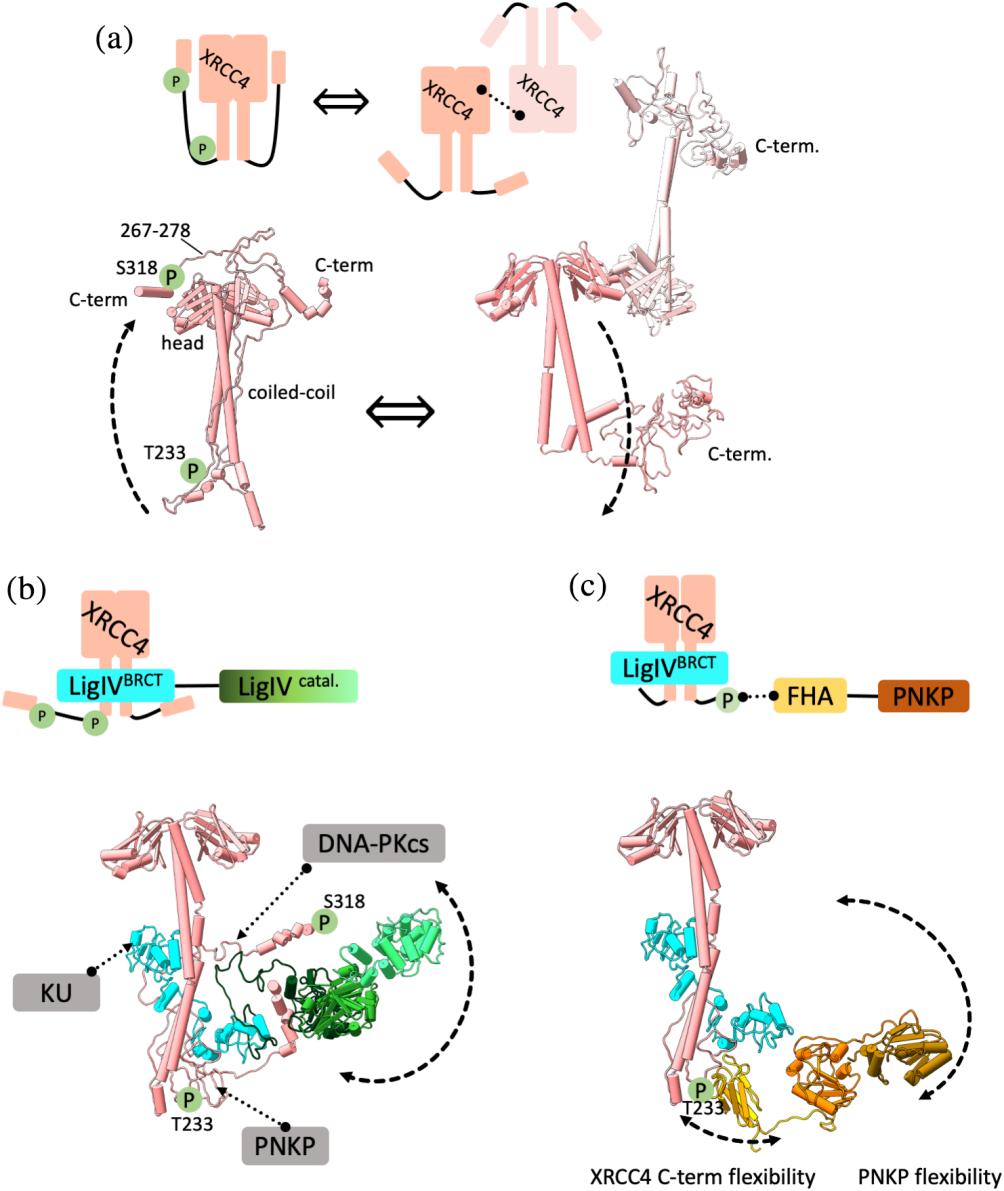
XRCC4 modular interactions, structural conformations, and dynamic assembly form the core for the NHEJ flexible scaffold. (a) XRCC4 schematic representation highlights a homodimer to tetramer transition that drives extension of the XRCC4 disordered C‐terminus. SAXS models of XRCC4 homodimer with folded back C‐terminus and XRCC4 tetramer with displaced C‐terminus (from Reference 99). (b) Schematic representation and SAXS model of XRCC4‐LigIV assembly showing LigIV catalytic core flexibility enabling the catalytic domains to be proximal to the tandem LigIV^BRCT^ domain (from Reference 43). Interacting regions of XRCC4 with partner proteins are indicated. (c) Schematic representation and SAXS model of XRCC4‐LigIV^BRCT^‐PNKP assembly highlight the flexibility of the PNKP catalytic core tethered to the disordered, phosphorylated XRCC4 C‐terminus by the FH domain (from Reference 117). (a–c) Complex components are colored according to the schematic representations. Solid and dotted lines represent flexible tethers or components interactions, respectively. CK2‐phosphorylation sites (S232 and T233) and DNA‐PKcs phosphorylation sites (S318, S260, and others) are highlighted with the green circles. XRCC4 C‐terminal region (267–278) that bind DNA‐PKcs FAT domains is indicated

Although cryo‐EM samples of LR synaptic complex contained XRCC4 complemented with full‐length LigIV, the LigIV catalytic domains were not visible (Figure 1c), reflecting their flexibility as directly indicated by SAXS results^25^ (Figures 2b and 4a). On the other hand, the disordered XLF C‐terminus^99^ reaches across to interact with Ku80.^10^, ^100^ Overall, the LR synaptic complex is formed by a DNA‐PK dimer supported by a “web”‐like tethers between XLF and Ku80; XRCC4 and DNA‐PKcs; and LigIV^BRCT^ and Ku80 (Figure 1c). Together these tethers form a flexible bridge that is also a linchpin for the complex due to protruding helical coiled‐coil interactions from XRCC4 and XLF with KU. Thus, the conserved but disordered C‐terminus of XLF and XRCC4 plays a crucial role in promoting DNA‐PK catalytic activities for NHEJ initiation.^10^ Notably, the LR complex holds, protects, and tethers the two DNA ends while retaining KU on the dsDNA.

## 4 AUTOPHOSPHORYLATION OF DNA‐PK DIMER ALLOSTERICALLY SWITCHES NHEJ CONFORMATIONS AND COMPLEXES TOWARD END PROCESSING AND ALIGNMENT

The dynamic integrated structures and knowledge of the auto‐phosphorylation sites enable dissection of DNA‐PK functions. ABCDE sites phosphorylated enabled Artemis catalytic activity^101^ and DNA‐PKcs release.^81^, ^102^, ^103^, ^104^, ^105^ Opened DNA ends are required to promote other DNA‐PK autophosphorylations and phosphorylation of other DNA‐PK targets. Recent studies data suggest that DNA‐PK activation proceeds through at least two distinct steps (Figure 1d).^106^ In the first step, assembly of Ku and DNA‐PKcs onto double‐stranded ends is sufficient to promote autophosphorylation of the ABCDE sites, which in turn activates Artemis can open DNA hairpins.^107^ Indeed, our previous SAXS experiments show more stable bridging of DNA‐PKcs dimer in the presence of DNA with separated DNA strands.^2^, ^81^ The structure of the LR complex shows that the DNA end‐blocking (DEB) helix (2,736–2,767) spans the large space cradled by the HEAT repeats separate 5′ from 3′ DNA ends and suggests a molecular mechanism for blocking DNA ends^10^ (Figure 1d). Whether the DEB helix stabilized overhang and hairpin DNA ends in the same matter as melted DNA blunt end or permit it further sliding in the space cradle is uncertain. However, the DEB helix is flanked by the unstructured ABCDE sites and the evolutionarily conserved YRPD motif, suggesting that the DEB helix coordinates the interactions between the DNA‐PKcs dimer and autophosphorylation of the ABCDE cluster. Thus, in the first step toward transition to SR synapsis, the ABCDE autophosphorylation may function as an electrostatic switch (see Figure 1d) that destabilizes the binding of DNA‐PKcs to DNA ends^104^ with the DEB helix functioning as ruler for the Artemis access to process the DNA ends. Notably, blocking autophosphorylation (changing identified sites to alanine) reduces nucleotide loss at coding joints in episcopal assays, and mimicking autophosphorylation (changing sites to Asp/Glu) increases the nucleotide loss at coding junctions validating this regulation as important in cells.^108^

In a subsequent step, the DNA strand separation by DEB helix, as shown in LR complex structure (Figure 1d), is required to promote PQR autophosphorylation and full kinase activation towards DNA‐PK's many substrates.^106^, ^109^, ^110^ Thus the DNA‐PKcs in the LR complex structure are likely to be active, allowing the autophosphorylation in trans of both ABCDE in the first step and PQR in the second step.^82^ The DEB helix is disordered in the structures of monomeric DNA‐PK in the absence of XLF‐XRCC4 flexible bridge and linchpin,^85^, ^87^ and this further supports the critical role of XRCC4‐XLF bridge in the DNA‐PK activation.^111^ Importantly, blocking autophosphorylation at these sites can reduce a cell's ability to utilize the HDR for DSBR emphasizing the connections between NHEJ and HDR. Whereas blocking phosphorylation at ABCDE sites inhibits both end processing and HDR, blocking PQR autophosphorylation enhances both^112^ suggesting that more structural analysis with SAXS may be important to define these distinct phosphorylation states.

PQR autophosphorylation and full kinase activation can phosphorylate XRCC4 C‐terminal tails that seem to be aid the release of DNA‐PKcs from LR and the switch to SR complex. XRCC4 C‐terminal region (267–278) bind the charged grooved formed by DNA‐PKcs FAT domains (Figure 1d) to tether DNA‐PKcs in the LR complex while likely guiding the disordered C‐terminal phosphorylation sites (S318, S260, and others)^113^ to the DNA‐PKcs kinase.^10^ We suggest that after the release of DNA‐PKcs, the XRCC4 disordered C‐terminal region interacts with PNKP,^114^ permitting further processing of DNA ends in SR synaptic complex. The phosphorylation‐dependent recruitment of PNKP to XRCC4 relies on a conserved forkhead‐associated (FHA) domain that binds and recognizes the disordered XRCC4 C‐terminus phosphorylated by CK2^114^, ^115^, ^116^ in a flexible and dynamic arrangement^117^ (see next section and Figure 2a,c). Thus, the mutation or truncation of the disordered XRCC4 C‐terminus, which disrupts both LR and SR complex arrangements, are associated with prenatal and postnatal growth failure and leukopenia^77^ and identified in the cancer mutation database.^118^

The two‐step DNA‐PKcs autophosphorylation outlined above is now a structurally and functionally validated electrostatic switch. Furthermore, once activated DNA‐PKcs phosphorylates many NHEJ proteins and sites. Yet the impact of this has been strikingly enigmatic and controversial.^119^ For example, Artemis is heavily phosphorylated by DNA‐PK, but assays have not shown that phosphorylation of these sites impact NHEJ.^107^ Also, ATM may phosphorylate these sites in cells.^120^ Similarly, blocking all DNA‐PK phosphorylation sites on XLF and XRCC4 has an impact on DNA bridging but only mild cellular phenotypes.^111^, ^121^, ^122^ LigIV is also phosphorylated without major assayed impact.^123^ On the other hand, KU phosphorylation can facilitate disruption of the complex and control DSB repair pathway choice.^124^ Unfortunately, the absence of impact in a given biological or biochemical assay may be informative, but it may not indicate an absence of important function as often inferred. Rather it shows that the tested component is not rate limiting in the particular assay being employed, which also may not consider avoidance of harmful activities and the need for coordination with other processes inside cells. Thus, for the NHEJ supramolecular machine and the NHEJ process, which we maintain is not a strictly linear pathway, we suggest that structural models can be invaluable to define assays that may optimally test the significance of DNA‐PK phosphorylation sites. In fact, this has directly been shown for DNA‐PKcs autophosphorylation, where in vitro assays show NHEJ without the DNA‐PK catalytic subunit DNA‐PKcs,^77^, ^78^ but structures uncover its key roles in coordinating and orchestrating initial NHEJ steps as noted below.

## 5 XRCC4 DYNAMIC INTERACTIONS AND ASSEMBLIES FORM THE CORE NHEJ FLEXIBLE BRIDGE

After protecting the two DNA ends in the LR synaptic complex, DNA‐PKcs kinase activity, along with XRCC4, XLF, and LigIV, are required to transition to a SR synaptic complex in which KU has aligned DNA ends for processing and ligation.^79^, ^80^, ^125^ Notably, as engagement of the DNA ends activates DNA‐PK activity,^126^ this provides a key checkpoint to ensure that there are two free DNA ends held in the complex with autophosphorylation in trans resulting in DNA‐PKcs release from DSB ends.^82^ Effectively this autophosphorylation provides an electrostatic switch to release DNA‐PKcs from the two DSB ends,^10^ analogously to electrostatic control of proteins for electron transfer.^127^

DNA‐PKcs activity triggers concerted conformational change by releasing the strain within the LR complex conformation for the LigIV–XRCC4–XLF–XRCC4–LigIV bridge as well as in XLF–Ku80^100^ and LigIV^BRCT^–Ku70^10^ interactions to align the DNA DSB ends for ligation. Strikingly, the DNA‐PKcs HEAT cradle region is suitable to act as a “ruler” in the LR complex for the appropriate length of DNA for subsequent alignment in the SR complex.

In the SR complex, two KU‐DNA complexes are aligned through a network of intermolecular interactions, where XRCC4 and XLF disordered C‐terminus are stabilizing the synaptic complex. XRCC4 can interact with itself to form multimers and filaments,^99^, ^128^ as well as with the tandem LigIV^BRCT^ domain, XLF, PNKP, APLF, and KU‐DNA.^10^, ^25^, ^43^, ^129^ The cryo‐EM complexes^10^ are consistent with individual structures of the XRCC4 homodimer,^130^ XLF homodimer,^131^, ^132^ LigIV catalytic core,^133^ PNKP,^116^ KU,^74^ APLF domains^134^, ^135^; and XRCC4 in complex with tandem LigIV^BRCT^ domain,^135^ or XLF^26^, ^98^, ^128^, ^136^ as solved by MX or NMR. SAXS was key to visualize and characterize XRCC4 multimers,^99^ flexible assembly with LigIV,^43^, ^99^, ^137^ PNKP,^117^ KU‐DNA‐APLF,^25^ and formation of XRCC4‐XLF filaments.^26^, ^99^ Importantly, the SAXS technical advances by measuring SAXS in line with size exclusion chromatography (SEC‐SAXS) allowed characterization of dynamic XRCC4 assemblies. SEC‐SAXS separates transiently self‐associating XRCC4 multimers from XRCC4 dimer and monomer.^99^ The solution state of the XRCC4 monomer shows a flexible C‐terminus, and suggests this C‐terminus is folded back and located nearby the N‐terminal head domain (Figure 2a).^99^ Together with the atomistic modeling, SAXS furthermore shows the formation of XRCC4 tetramer via a head‐to‐head interface and further suggests a release of the C‐terminus from the N‐terminal head region^99^ (Figure 2a).

Interestingly, SAXS measurements also show that XRCC4 multimers are disrupted when the tandem LigIV^BRCT^ domain encircles the XRCC4 coiled‐coil region^43^, ^99^, ^137^ followed by releasing the XRCC4 C‐terminus from the N‐terminal head region^99^ (Figure 2b). Given that human cells contain more XRCC4 than LigIV,^138^ it seems unlikely that each subunit of XRCC4 contains a bound LigIV molecule. Therefore, XRCC4 multimers may represent a transient storage form^99^, ^128^ that dissociates into homodimers upon interaction with the LigIV (Figure 2b). Release of C‐terminus upon LigIV binding may function as a conformational switch that permits interaction of tandem LigIV^BRCT^ domain with KU to further stabilized synaptic complex^10^ (Figures 1c and 2b). Thus, conformational plasticity of the XRCC4 C‐terminus plays an essential role in the transition between LR and SR synaptic complex.

Similarly, flexibility of the LigIV catalytic core plays a critical role in the progression of NHEJ. Although the cryo‐EM structure of XRCC4‐LigIV positions the catalytic domain of LigIV near the XRCC4 head domain,^139^ SAXS indicates that the flexible LigIV catalytic core domains are in proximity to the tandem LigIV^BRCT^ domain (Figure 2b). SAXS furthermore uncovers the conformational variability between the individual catalytic domains of LigIV,^43^, ^137^ a distinctive property of all human DNA ligases that permits the catalytic domains to encircle the DSB.^1^, ^140^, ^141^, ^142^

The disordered XRCC4 C‐terminus facilitates its CK2‐phosphorylation controlling PNKP recruitment^117^ essential to process DNA termini^143^ for subsequent ligation by the LigIV^144^ (Figure 2c). The PNKP (3′‐DNA phosphatase, 5′‐DNA kinase) replaces non‐ligatable groups at DNA termini with ligatable 5′‐phosphates and 3′‐hydroxyl groups.^143^ Combining the PNKP crystal structure with SAXS analyses of PNKP reveals a flexible tether between the N‐terminal fork‐head associated (FHA) domain and catalytic phosphatase‐kinase domain.^116^, ^145^ The FHA domain interacts with CK2‐phosphorylated XRCC4^146^ through a phosphorylated site in the disordered XRCC4 C‐terminus^117^ (Figure 2c). Advances in the SEC‐SAXS technique permitted visualization of a transient XRCC4‐LigIV‐PNKP complex showing that stable PNKP binding to XRCC4‐LigIV complex requires XRCC4 S232, T233 phosphorylation and that only one PNKP protomer binds per XRCC4 homodimer.^117^ SAXS‐based dynamic assessment of the purified complex suggests flexible tethering of PNKP to disordered XRCC4 C‐terminal region via the FHA‐phosphopeptide interaction. Overall, SAXS multistate models indicate that the complex can adopt compact and extended conformations: these imply dynamic interactions between PNKP catalytic domain and XRCC4 head region or the tandem LigIV^BRCT^ domain^117^ (Figure 2c). Combined flexible tethering between PNKP catalytic domain, FHA, and disordered XRCC4 C‐terminal region allows PNKP catalytic domain to be far‐reaching to process DNA ends without disrupting the SR synaptic complex.

## 6 APLF DISORDER AND MODULAR INTERACTIONS ADD STABILITY TO THE FLEXIBLE NHEJ SCAFFOLD

**FIGURE 3:**
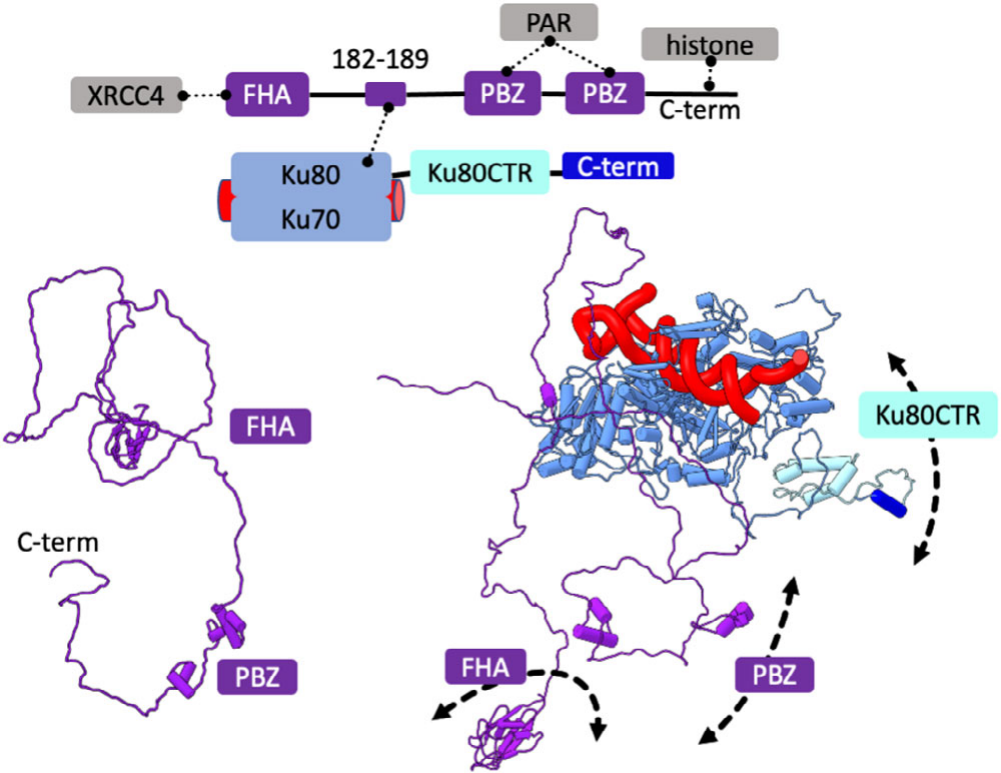
APLF disordered regions and modular interactions contribute to the flexible NHEJ scaffold. Top panel: Schematic representation of APLF‐KU‐DNA complex with highlighted interacting regions of partner proteins. Bottom panel: SAXS model of APLF in the free and complexed state (taken from Reference 25). SAXS models highlight APLF disorder suitable to support recruitment of XRCC4, PAR‐modify proteins; and anchor NHEJ complex to neighboring nucleosomes. Complex components are colored according to the schematic representation. Solid and dotted lines represent the flexible tethers or components interactions, respectively

APLF has emerged as an added scaffolding protein in NHEJ. APLF interacts with phosphorylated XRCC4 via its N‐terminal forkhead associated (FHA) domain^146^, ^147^ while interacting with Ku80 via its mid‐domain^100^, ^148^, ^149^ and poly‐ADP ribose modified proteins via its C‐terminal PAR‐binding zinc finger (PBZ) domains^135^, ^150^ (Figure 3). The APLF in solution is an intrinsically disordered protein with embedded locally structured interaction regions (Figure 3). These mediate interactions with KU‐XRCC4‐LigIV complexes on DNA ends, whereas XRCC4‐LigIV bridges DSB ends between adjacent KU molecules (Figure 3).^25^

**FIGURE 4:**
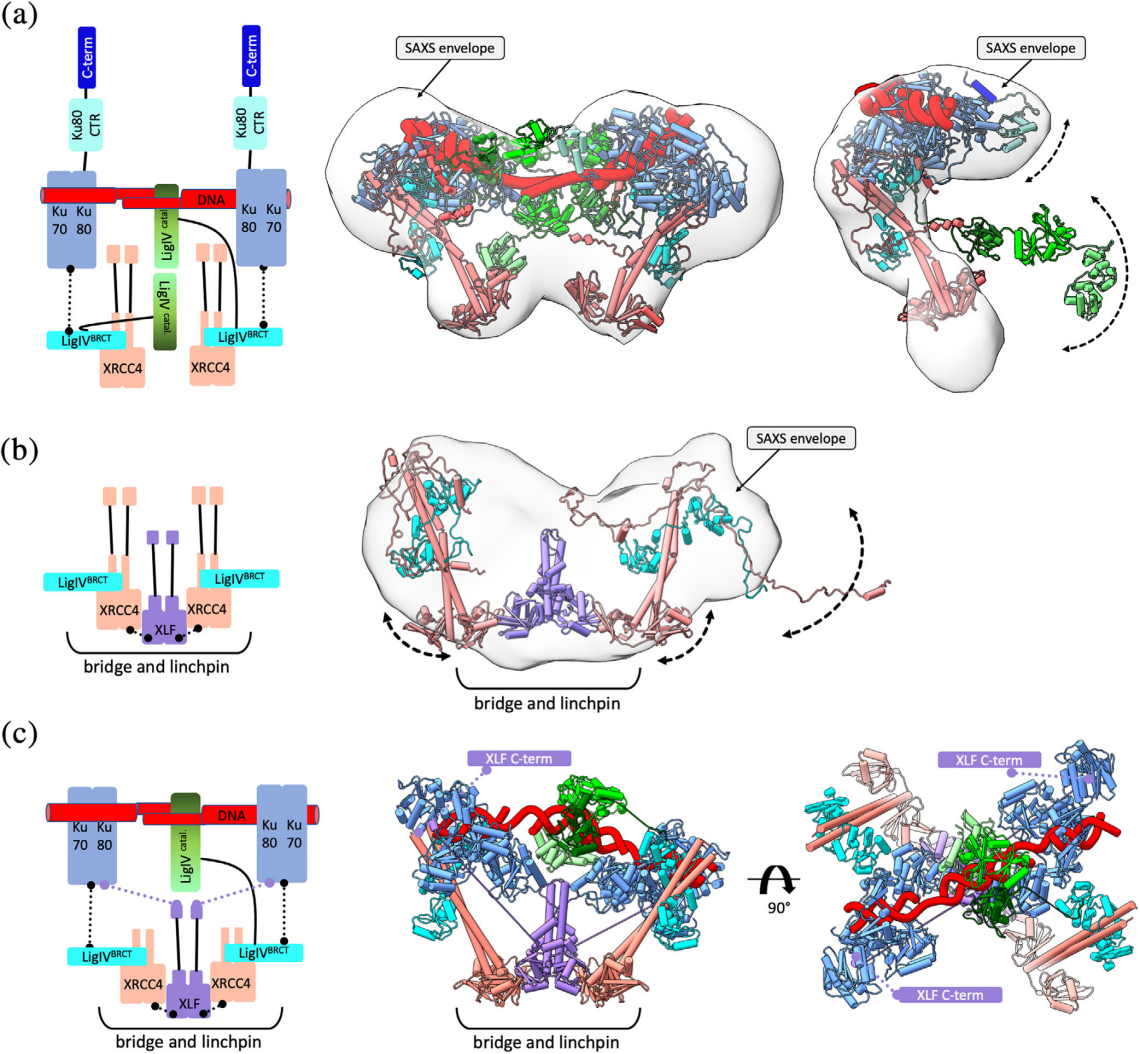
Toward an atomic structure for the short‐range synaptic complex. XRCC4‐XLF forms a bridge and linchpin to LigIV and KU to position and stabilize the short‐range synaptic complex. (a) Left panel: Schematic representation of the synaptic KU‐XRCC4‐LigIV complex bridged by DNA containing long overhang. Middle panel: SAXS envelope (gray) of the KU‐DNA‐XRCC4‐LigIV‐APLF complex indicates the location of KU with the XRCC4‐XLF linchpin and XRCC‐LigIV relative to the aligned and annealed DNA overhangs. Right panel: SAXS envelope (gray) of the KU‐20 bp DNA‐XRCC4‐LigIV‐APLF complex superimposed with the atomistic models of its components. The mismatch between the SAXS envelope and atomistic model suggests the presence of disordered APLF and the flexibility LigIV catalytic domain. Note that atomistic models do not include APLF due to its disorder (from Reference 25). (b) Left panel: Schematic of the XRCC4‐LigIV^BRCT^‐XLF complex derived from the SAXS model shown in the right panel. Right panel: SAXS envelope (gray) of the XRCC4‐LigIV^BRCT^‐XLF complex and the proposed atomistic model highlight conformational plasticity of the XRCC‐XLF‐XRCC4 bridge and the disordered character of the XRCC4 C‐terminus (from Reference 99). (c) Schematic and two orthogonal views of the cryo‐EM structural model of the SR synaptic complex.^10^ The extension of the XLF C‐terminus is highlighted. (a–c) Complex components are colored according to the schematic representation. Solid and dotted lines represent the flexible tethers or components interactions, respectively

As the KU‐XRCC4‐LigIV complex stimulates ligation, and this complex is stabilized by APLF,^25^, ^148^ the KU‐DNA‐XRCC4‐LigIV‐APLF scaffolded assembly may aid DNA ligation during DSB repair in vivo.^151^ SAXS solution state modeling shows that APLF remains disordered upon complexation with KU (Figure 3). The flexible APLF N‐terminal FHA domain in KU‐DNA‐APLF assembly may further promote interaction with XRCC4‐LigIV. Indeed, our solution studies confirm the stabilization of the KU‐XRCC4‐LigIV complex in the presence of APLF.^25^ SAXS data determined the dimensions and shape of the complex assembled on the short 20 bp DNA. SAXS measurements indicate a multinodular, elongated assembly with a 1:1:1:1  ratio (Figure 4a, right panel). The relative position of the XRCC4‐LigIV and Ku‐DNA components was determined using a multiphase SAXS envelope.^152^ The arm‐like protrusion located at the far extremity of the SAXS model suggests flexible‐tethering of the LigIV catalytic core (Figure 4a, right panel). However, the flexible APLF C‐terminal PBZ domains lacking the binding PAR‐modified partner^135^, ^150^ did not permit accurate localization of APLF. We suggest however that the disordered APLF C‐terminus may facilitate contact with histones to stabilize the synaptic complex in the context of the neighboring nucleosomes.

On the other hand, the SAXS envelope of the KU‐XRCC4‐LigIV‐APLF complex formed with two DNA's with complementary overhangs shows two oppositely positioned bulky regions and two central located protrusions (Figure 4a, left and middle panel). Superimposing the atomistic models of the complex components with the SAXS envelope suggests the overall architectural arrangement of the synaptic KU‐DNA‐XRCC4‐LigIV complex^25^ (Figure 4a). The XRCC4‐LigIV is located in the center of the assembly and links two external KU‐DNAs with the DNA aligned close to the XRCC4‐LigIV interface (Figure 4a). This arrangement is consistent with the proposed model from EM projections^153^ and provided insights to guide reconstructions of cryo‐EM's high‐resolution structure of the SR NHEJ complex (Figure 4c).^10^ The reconstructed solution model lacks the resolution of cryo‐EM structure; however, it shows synaptic complex formation through DNA bridging in the absence of XLF. This strategy was further explored in selecting a DNA substrate with a long overhang to stabilized SR synaptic complex for the cryo‐EM study.^10^ Notably, these solution studies also show that APLF itself is not sufficient to stabilize the SR synaptic complex (Figure 4a, right panel).

## 7 XLF FORMS A CENTRAL LINCHPIN FOR THE SYNAPTIC LIGATION COMPLEX

XRCC4 interacts with the structurally related XRCC4‐like factor (XLF),^154^, ^155^ which stimulates the activity of LigIV toward non‐compatible DNA ends in vitro^156^, ^157^ by promoting re‐adenylation of LigIV.^97^ XLF consists of a globular head domain, an elongated coiled‐coil stalk,^131^, ^132^ and a disordered C‐terminal region^99^ that interacts with Ku80^10^, ^100^ (Figure 4d). Combined crystallography and SAXS show that the XRCC4 head domain forms a hydrophobic pocket for specific interaction with the XLF head domain via L115.^26^, ^98^, ^128^, ^136^ When XRCC4 is in complex with LigIV, the XLF can bridge two XRCC4‐LigIV complexes.^99^ The reconstructed SAXS envelope of XRCC4‐LigIV^BRCT^‐XLF shows two elongated regions, consistent with two XRCC4‐LigIV^BRCT^ separated by a central protrusion attributable to XLF (Figure 4b).^99^ The plasticity between the XRCC4 and XLF head domain contacts^26^, ^98^, ^99^, ^136^ may lead to an even more significant separation of the two XRCC4‐LigIV^BRCT^ molecules (Figure 4b). The adaptable XRCC4 separation allows flexible bridging of KU‐DNA, as further suggested by the weak electron densities map of the XRCC4‐XLF region in the cryo‐EM structure of the LR and SR synaptic complex^10^ (Figure 4c).

SAXS suggests that KU can bind DNA ends within the XRCC4‐LigIV assembly^25^ and be stabilized through the interactions between Ku80 and LigIV^BRCT^,^10^ whereas the LigIV catalytic core is flexibly linked to the LigIV^BRCT^ ^43^, ^137^ (Figure 4a). In this specific integrated model, the distribution of LigIV delivers a capacity for repositioning the DNA ends,^158^ promoting efficient end‐to‐end configuration and ligation. How such complexes allow end processing may depend upon their flexible attachments. The LigIV catalytic domain's adjustable extension is achieved by tethering to the XRCC4‐XLF‐XRCC4 bridge (Figure 4), where flexible XLF C‐terminus additionally tether Ku80^10^, ^100^ to keep DNA ends nearby. Thus, the XRCC4‐XLF‐XRCC4 bridge acts as an adjustable DNA tether: it flexibly connects the LigIV catalytic region for its recruitment to the properly positioned DNA ends yet also stabilizes the SR complex by acting as a flexible linchpin to LigIV and Ku (Figure 4c).^10^ Even with substrates containing two nicks, only a single LigIV catalytic domain was visible within the SR complex's cryo‐EM structure (Figure 4c), supporting the single turnover activity of LigIV.^97^, ^159^ Thus, two LigIV must sequentially join both strands of the DSB.

## 8 XLF‐XRCC4 FILAMENT FORMS A SUPER‐HELICAL CHANNEL FOR OVERALL ALIGNMENT OF DNA ENDS

**FIGURE 5:**
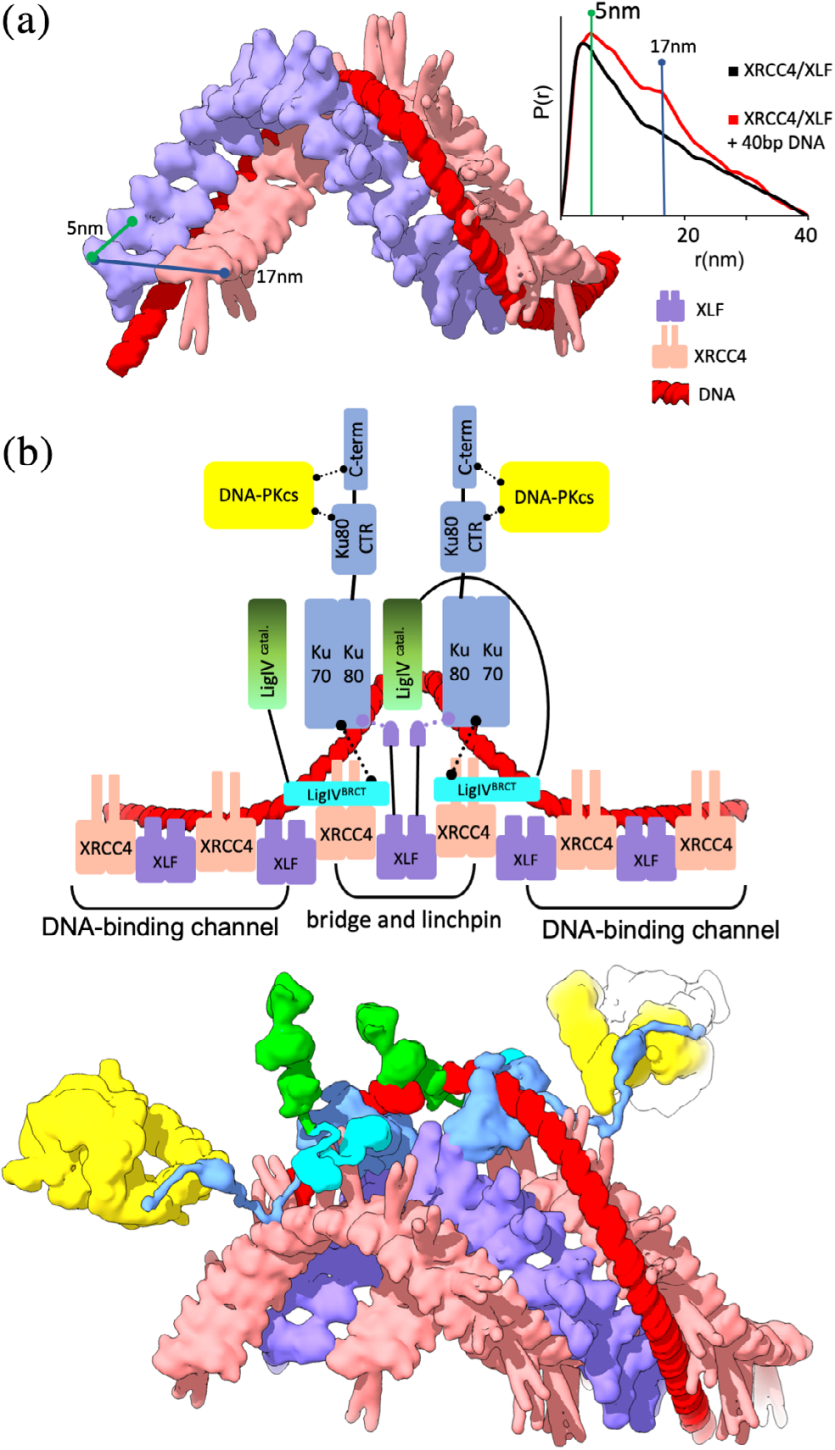
Combined X‐ray crystallographic and SAXS structures explain the synergy of XLF‐XRCC4 grooved scaffold and synaptic complex. (a) Super‐helical channel of XLF‐XRCC4 molecular surface. The parallel XLF‐XRCC4 unit is shown as seen in the crystal structure.^26^ DNA positioned based on HDX and docking experiment (from Reference 26). Components of the super‐helical channel are colored according the legend. Left panel: Experimental pair distribution function [P(r)] of XLF‐XRCC4 and XLF‐XRCC4‐40 bp DNA (from Reference 99) highlight the intramolecular distances between XLF and XRCC4 marked in the molecular surface. (b) Synaptic complex nucleated XLF‐XRCC4 filament appears suitable to maintain DNA end alignment via its grooved DNA‐binding surface (modified from Reference 26). This theoretical model, which utilizes the filament groove, is distinguished by having a synaptic complex proximal to the DSB, allowing possible DNA‐PK activation of partners, and providing steric access to processing enzymes and LigIV with the DNA. Complex components are colored according to the schematic representation shown in the top panel. Solid and dotted lines represent the flexible tethers or components interactions, respectively

While the XLF‐XRCC4 complex directly bridges KU and LigIV in the SR synaptic complex,^10^ there are supramolecular models for its role in larger‐scale assemblies holding dsDNA adjacent to DSBs due to its ability to form channeled filaments. For HDR repair of DSBs, RAD51 filaments protect DNA end regions are a known key feature of HDR repair. Even short RAD51 filaments are important to avoid stalled replication fork degradation by the MRE11 nuclease.^160^ Interestingly combined crystallographic and SAXS data show that the XLF‐XRCC4 interaction through their head domains can form super‐helical filaments suitable to help protect regions flanking DNA ends and support their architectural placement for ligation (Figure 5).^26^, ^98^, ^99^, ^128^ In fact, XRCC4‐XLF filaments, which are further stabilized in the presence of DNA, create an extended grooved channel with the potential to align DNA end regions to facilitate the formation and further stabilization of the SR complex for ligation^26^ (Figure 5a). Furthermore, these XLF‐XRCC4 filaments have been proposed to be important for repair in cells.^129^, ^158^

As discussed above, in the SR synaptic complex XRCC4 interactions with LigIV disrupt XLF‐XRCC4 filaments (Figure 4b). This suggests two different possible roles of XLF in the final steps of NHEJ.^99^ A synergistic model of filaments and synaptic complex for the NHEJ ligation was proposed.^25^, ^158^, ^161^ The grooved channel formed by XRCC4‐XLF filaments can guide dsDNA but also support positioning LigIV for catalysis. High‐resolution imaging in cells is consistent with XRCC4‐XLF filaments forming “sliding sleeves” around and over KU bound at DSBs^161^ (Figure 5b). The breakthrough cryo‐EM structure of the synaptic complex provides a possible mechanistic basis for the Ku‐DNA‐dependent recruitment of the XLF‐XRCC4‐LigIV complex through the network of flexible tethers.^10^ Notably, the head‐to‐head interface between XLF and XRCC4 dimers resembles that observed in the filamentous structure.^26^ XLF–XRCC4 filaments stabilize dsDNA adjacent to the DSB whereas the linked DNA‐PK LR complex tethers the DSB ends. We reason that the synaptic complex may remain bound to the DNA termini in concert with the grooved XRCC4‐XLF binding channel (Figure 5b)^26^, ^98^, ^128^, ^158^ flanking the synaptic complex.

In the initial NHEJ step, Ku recruits DNA‐PKcs to DNA ends.^162^, ^163^ Upon recruitment, DNA‐PKcs undergo auto‐phosphorylation‐dependent conformational changes that release DNA‐PKcs, enabling remodeling of the XLF‐XRCC4 bridging linchpin to support the protection of DNA ends. The timing of DNA‐PKcs recruitment and release, coordinated with the formation of synaptic complex and construction of the XRCC4‐XLF DNA‐binding channel, are all unknown. Interestingly, both DNA‐PKcs and XLF appear to protect DNA ends from resection.^164^, ^165^, ^166^, ^167^, ^168^ Thus, the XRCC4‐XLF DNA‐binding channel may form after DNA‐PKcs has been released from DNA ends and, at this point, the XLF‐XRCC4 filaments may function to restrict DNA end resection (Figure 5b). DNA‐PKcs displacement from the DSB at the ligation stage (Figure 5b) provides potential mechanistic insight into in vivo studies showing that auto‐phosphorylation of DNA‐PKcs is necessary to relieve the physical blockage on end‐ligation imposed by the DNA‐PKcs protein itself.^169^ Thus, the DNA‐PKcs auto‐phosphorylation and consequent electrostatic switch enable NHEJ to maintain its flexibly bridged assembly as XRCC4‐XLF provides a flexible bridge and linchpin to both Ku and LigIV while enabling geometric access of enzymes such as PNKP to the DNA ends.^10^ The capacity of XRCC4‐XLF to form a DNA‐binding channel flanking the ends may help position and protect the DNA end regions from resection^161^ (Figure 5b), but this remains incompletely understood.

## 9 ENVISIONING THE MECHANISM FOR THE MULTI‐COMPONENT NHEJ MACHINE

How does NHEJ, which is more like a multi‐component, multifunctional machine than a linear pathway,^64^ function mechanistically for its coordinated movements, assembles, and regulation? The observed switch from a DNA‐PKcs central dimer in the LR complex to more distally placed flexible DNA‐PKcs monomers linked to the KU‐XRCC4‐XLF flexible scaffold in the SR synaptic complex unveils the structural basis for NHEJ functional coordination and regulation. Although not technically required for NHEJ activity, the initial DNA‐PKcs dimer provides critical end protection and temporal coordination for the core XRCC4‐XLF bridge and scaffold assembly consistent with its evolutionarily conserved YRPD motif.^44^ Notably ATM may be able to phosphorylate DNA‐PKcs ABCDE sites in vitro; however, the DNA‐PKcs dimer structure geometrically restricts possible ATM access at a two‐ended DSB, so in cells ATM phosphorylation would likely only occur in a backup pathway when the functional DNA‐PKcs dimer is somehow disrupted. This point emphasizes the importance of negative design that prevents disruptive and conflicting pathway interactions in vivo and needs to be considered in devising assays to test the functional roles of components of molecular machines.

Upon DNA‐PKcs dimer disassembly, the DNA ends can be aligned and moved together for ligation. Yet, the resulting dynamically tethered DNA‐PKcs allows targeted phosphorylation of other NHEJ proteins without disrupting the SR synaptic complex. Longer range dynamic pairing of end‐to‐end DNA in vivo^161^ and in vitro^158^ through XRCC4‐XLF DNA‐binding channel^26^, ^98^, ^128^, ^136^ would seem prohibited in the LR complex with DNA‐PKcs and KU located at the DNA ends. In the SR synaptic complex integral to the tethering and ligation of DSB ends,^10^ KU80CTR connects DNA‐PKcs through a flexible attachment,^76^, ^85^, ^86^, ^87^ so LigIV and PNKP can carry out their enzymatic functions at DNA ends. Therefore, the flexible scaffold‐like arrangement of BRCT in LigIV and the FHA in PNKP suggest mechanisms to control these enzymes' access to DSB ends rather than placing them throughout the assembled XLF‐XRCC4 filaments.

Machines need movement to function, and SAXS provides an objective assessment of movement including shape‐shifting transformer changes that enable the adaptable complementarity and super efficiency of biological nanoscale machinery. SAXS measurements can objectively examine structural similarity to assess biomachine movements, conformations, complexes. Yet, the recent innovation and speed of collecting SEC‐SAXS from solutions containing various NHEJ complexes in high‐throughput mode have yet to be fully exploited. We argue that this capability is becoming even more powerful given that the required screening of multiple conditions and component mixtures for cryo‐EM or MX to determine high‐resolution structures. Thus, identifying optimal component mixtures or buffer conditions for an atomic‐resolution structural technique makes SEC‐SAXS, which can be performed in under 30 min, increasingly valuable. To illustrate this, we show here global conformational comparisons by structural similarity map (SSM)^170^ as an analytical tool that discriminates and quantifies complexation and conformational similarities and differences among many different NHEJ complexes.

**FIGURE 6:**
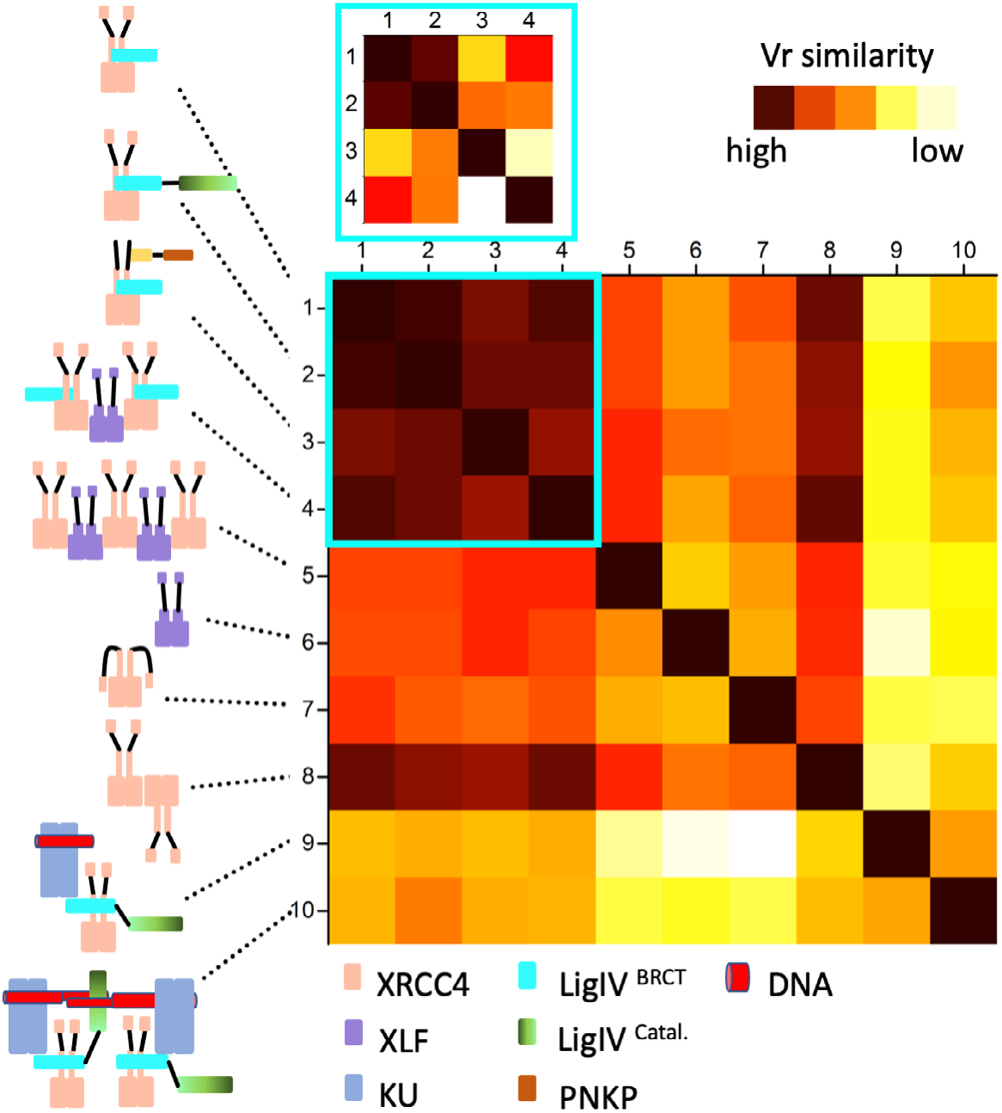
Structural similarity map (SSM): a tool to objectively measure NHEJ complexation by SAXS. A measure of structural similarity between experimental SAXS curves of XRCC4‐LigIV^BRCT^ (1), XRCC4‐LigIV (2), XRCC4‐LigIV^BRCT^‐PNKP(3), XRCC4‐LigIV^BRCT^‐XLF(4), XRCC4‐XLF(5), XLF(6), XRCC4 homodimer (7), XRCC4 homotetramer (8), XRCC4‐LigIV‐APLF‐KU‐20bpDNA(9), XRCC4‐LigIV‐APLF‐KU‐20bpDNA with overhang (10). The similarity was scored by the volatility of ratio (Vr).^170^ Scores were assigned a gradient color with a red—high agreement and white—low agreement. Inset: SSM of the first four complexes is shown. Components of complexes are colored according to the legend

The volatility of ratio (Vr) difference metric provides a quantitative and superposition‐independent comparative evaluation of structural similarity from many SAXS data sets.^170^ The results can be illustrated by plotting a diagonally symmetric heat map in which each matrix element quantifies the pairwise agreement between two of the SAXS data sets, color mapped from red (similar) to white (different) (Figure 6). However, the method provides quantitative numbers as well as the visualization shown here. The Vr values displayed in a heat map derive from the normalized ratio between two SAXS curves. For example, the heat maps show significant differences between XRCC4 complexes with LigIV present or absent. Notably, SSM also indicates the level of objective dissimilarity between XRCC4‐LigIV^BRCT^, XRCC4‐LigIV, XRCC4‐LigIV^BRCT^‐PNKP, and XRCC4‐LigIV^BRCT^‐XLF (#1–4), which is distinguishable when the significantly larger assemblies formed with KU (#9, #10), which are not included in the SSM (Figure 6, inset). SSM furthermore reveals the significant dissimilarity between XRCC‐XLF filament (#5) and its free components (XLF homodimer [#6], XRCC4 homodimer [#7], or XRCC4 homotetramer [#8]).

Taken together with existing structural and biological data, the SAXS defined flexible NHEJ complex conformations, architecture, and dynamic interface switching appears to constitute an appropriate supramolecular biological machine to facilitate the activities of and transitions between DSB recognition, processing, pairing, and ligation without a need to release the potentially toxic and mutagenic dsDNA ends prior to ligation. More generally, these data establish the abilities of SAXS SSM, as enabled by the recent capability of synchrotron SAXS, to screen multiple NHEJ component mixtures in solution, to provide resolutions sufficient to distinguish conformational states and to objectively characterize flexible assemblies in high throughput. We anticipate these SAXS technologies will be a major enabling resource for the structural biology of dynamic complexes, such as those acting in NHEJ.

## 10 EMERGING INSIGHTS, PERSPECTIVES, AND PROSPECTS

As NHEJ is the primary DSBR process in human cells, it is important to fully understand its mechanism including different levels of structural regulation that are emerging by combining biophysical and cellular results. Even in G2 cells, about 80% of X‐ray‐induced DSBs are repaired with fast kinetics by NHEJ.^171^ Moreover, NHEJ reveals exemplary key roles for modular interfaces that accommodate and require significant dynamics and disorder for their functions. The concept of keystone complexes that promote kinetically stable assembles, which first emerged from HDR,^172^ has recently advanced most in NHEJ complexes due to integrated SAXS, MX, and cryo‐EM structures. In fact, the NHEJ assembly forms a keystone complex linking DSBR machinery with immune development and innate immunity. Combined data shows that the XRCC4‐XLF flexible bridge and linchpin provide the critical dynamic scaffold to hold KU and LigIV, which position and join the dsDNA ends. We also know that the XRCC4 function in DSBR is important in normal development.^173^ Yet, XRCC4 also interacts with retinoic acid‐inducible gene I (RIG‐I), a key cytosolic RNA sensor that recognizes RNA virus and initiates the MAVS‐IRF3‐type I IFN signaling cascade. RIG‐I is recruited to DSBs, where it binds XRCC4 and suppresses virus integration into the host genome by preventing NHEJ.^174^ Thus, XRCC4 dynamic interfaces play critical roles in balancing DSBR and the host innate immune response against viruses.

Dynamic structural transitions are key features of NHEJ complex mechanisms for regulation and biological function. Although the existence of unstructured regions in NHEJ complexes has been appreciated for decades, we are only now able to establish objective quantitative models for their structures. Yeats asked, “How can we know the dancer from the dance”?^175^ This insightful, poetic question highlights the intimate connections of DSB components to their choreography, as also noticed for homologous recombination (HR) repair.^18^ So, we can best understand NHEJ when we integrate rather than separate component proteins from their interactions and coupled movements. By combined solution and atomic structural methods, we are only now being able to understand NHEJ components and complexes in terms of their choreographed structured and disordered regions, dynamic interfaces, and movements. In fact, the combination of SAXS measurements plus atomic structures enables a detailed and fundamental understanding of functional inter‐relationships joining folded and unstructured components to enforce protein conformations positioning DNA ends to protect the DSB and then to align and ligate the two ends.

Here by envisioning both NHEJ dancers and their dance, we now better understand the functional choreography for the major DSBR process in human cells. Collective data suggests that DNA‐PKcs are not essential for NHEJ. Yet, like PARP in single‐strand break repair, DNA‐PKcs makes NHEJ far more efficient while also serving as an effective barrier to prevent inappropriate HDR and to specifically license NHEJ. KU plus XRCC4‐LigIV are necessary and sufficient to achieve a flexible synapsis of blunt DNA ends, whereas these components alone cannot. The addition of XLF causes a transition to the SR complex, and maximum efficiency of synapsis is achieved quickly, supporting the flexible XRCC4‐XLF bridge and linchpin idea proposed here. An open question concerns how the dynamic NHEJ complex accommodates functional access for the NHEJ nuclease Artemis. We know, for example, that MRE11 nuclease is important for licensing HDR and can help align DNA ends for alternative end‐joining.^69^, ^171^ Interestingly, Artemis binds to both DNA‐PKcs and LigIV: it may be activated by DNA‐PKcs and then stays linked to LigIV.^106^, ^176^, ^177^ It will likely be important to visualize the dynamic architectural association of Artemis and possibly other nucleases such as WRN^68^ in NHEJ complexes. Notably, SAXS biophysical measurements describe dynamics and take us beyond static structures. SAXS measures surprising conformational changes in flexible systems that enable specificity, as seen by the XRCC4‐XLF flexible linchpin and bridge. In SAXS experiments, we find that folded domains provide anchors that reduce conformational search by attached disorder regions. This combination of folded and disordered regions enhances efficiency for inducible conformations and enables NHEJ complexes to direct a cascade of conformational transitions as seen in the changes from LR to SR synaptic complexes.

In general, DNA repair is the focal point for cellular regulation during DNA replication stress, development, differentiation, and responses to environmental damage. For example, the poly‐ADP ribosylation (PARylation) response to DNA breaks is linked to program cell death by an apoptosis‐inducing factor^178^ and to regulating innate immune responses, so viral enzymes removing PARylation are an antiviral target.^179^ Thus, structure‐based inhibitors can probe DNA repair and its interconnections for cell biology as well as provide foundations for potential drugs. Inhibiting DNA repair may trump direct DNA damage for biological and therapeutic impact, for example, although cadmium damages DNA, its major impact on genomic instability results from its inhibition of DNA mismatch repair.^180^ Importantly, flexibility and allostery as identified here in NHEJ complexes can be targeted for DR inhibitors.^181^, ^182^ Inhibitors of poly‐ADP ribose polymerase (PARP), which aids break repair, are successful against cancer by trapping PARP on damage and blocking repair^182^ and inhibitors of the glycohydrolase that removes poly‐ADP ribose and releases PARP1 are under active preclinical cancer investigation.^9^ Inhibitors can even mimic enzyme interactions with damaged DNA^183^ and drive protein instability as well as blocking activity.^34^, ^184^

The recognition of functional liquid–liquid phase transitions and macromolecular condensates mediated by unstructured protein regions and RNA provides an emergent added functional area for both DNA repair and integrated structural biology. Unstructured and multivalent protein and RNA components, such as those acting in NHEJ scaffolding, as well as PARylation promotes such transitions at DNA damage sites.^50^ So multiscale structural methods, such as SAXS, enable an emerging area of qualitative analyses inside condensates with new insights on the structural nature and mechanisms for forming and disassembling functional phase transitions^8^, ^42^ that can promote NHEJ assemblies and activities.^54^ We find that specificity is encoded in disordered regions by sequence motifs and that reversible multivalent activity forms phase condensates with rich biophysics and biochemistry to uncover. Liquid–liquid phase transitions not only concentrate some molecules but also exclude others and can change the reaction equilibrium and physical properties plus enhance scaffolding and regulation.

The keystone complexes, multifunctional components, macromolecular machine, specificity encoded in disordered regions by sequence motifs such as YRPD, and negative design concepts plus the principle of flexible conformational control with ordered regions anchoring disordered elements as presented here for NHEJ offer emerging insights into nanoscale controls of cellular outcomes to endogenous and exogenous stress, such as DSBs. In particular, the dynamically assembled NHEJ machine, which acts in a concerted cascade of events, can function without some parts, even DNA‐PKcs which have both scaffolding and kinase functions. In terms of linear pathway thinking, this would indicate the unimportance of DNA‐PKcs because if DNA‐PKcs were important in a linear pathway then subsequent steps could not occur without it. Here we maintain that DNA‐PKcs, which is relatively unimportant in a linear pathway model, is instead a master regulator in a machine model: it is important for dynamic scaffold for recognition of two DNA ends, kinase activation and phosphorylation, and switching to the SR synaptic complex. Without these multiple DNA‐PKcs functions, NHEJ results in more toxic and mutagenic chromosomal fusions, where a dsDNA end from a stalled replication fork or break may be joined to another chromosome site. A practical implication is that such multifunctionality is best studied with separation‐of‐function mutants or inhibitors rather than genetic knockout or depletion methods. So in our NHEJ machine concept, removing parts of the machine does not block product production of DNA end joining but instead creates a less efficient and less regulated process. However, inhibiting the movement of active parts, for example, by inhibiting DNA‐PKcs, will block functional outcomes, which is exactly what combined data shows.^185^, ^186^, ^187^, ^188^ In terms of kinetic efficiency, most DSBR events can occur quickly by NHEJ, so we can envision that dynamic DNA‐PKcs complex assembly is important for efficient, rapid repair by NHEJ and that the homology dependent repair MRE11 complex is capable of removing assembled DNA‐PK complexes if they have not engaged in productive repair^189^ to initiate HDR and provide a means of biological pathway choice.^171^

Looking ahead, we expect that future studies will employ ongoing SAXS advances to increasingly focus on NHEJ molecular mechanisms and how the NHEJ machine and its components function and are interconnected with phase transitions, RNA, innate immunity, DNA replication, and resistance to radiation therapy. For example, the newly identified Survivin‐DNA‐PKcs heterotetramer complex and its impact of DNA‐PK dependent radiation survival will be of interest for structural and inhibitor analyses to inform cell biology and cancer therapeutics.^190^ Dynamic structures that reveal multifunctionality will be key to complement depletion studies where all functions are removed together with many resulting compensatory changes in cells. Guided by structures and multifunctionality, it will be exciting to employ SAXS to learn how to best target conformational transitions with chemical inhibitors that can trap repair intermediates analogously to PARP1 and poly(ADP‐ribose) glycohydrolase (PARG) inhibitors that trap PAR‐complexes to selectively kill cancer cells with low toxicity to normal cells. Overall, these combined structural methods provide a pathway to define and test dynamic structures of functional protein complexes with their biologically important RNA and DNA partnerships.

## AUTHOR CONTRIBUTIONS

**Michal Hammel:** Conceptualization; writing‐original draft; writing‐review & editing. **John A. Tainer:** Conceptualization; writing‐original draft; writing‐review & editing.
